# Sentinel node procedure in Ib cervical cancer: a preliminary series

**DOI:** 10.1054/bjoc.2001.2005

**Published:** 2001-09

**Authors:** T Lantzsch, M Wolters, J Grimm, T Mende, J Buchmann, G Sliutz, H Koelbl

**Affiliations:** Departments of Gynecology, Nuclear Medicine, Pathology, Martin-Luther-University, Halle/Saale, D-06097, Germany; Department of Gynecology, University of Vienna, Vienna, A-1090, Austria

**Keywords:** sentinel lymph node, cervical cancer, lymphoscintigraphy, lymphatic mapping

## Abstract

The aim of this study was to determine the diagnostic accuracy and feasibility of sentinel lymph node (SLN) detection using a gamma probe in patients with Figo IB cervical cancer. Between January 1999 and September 2000, 14 patients with cervical cancer, planned for radical hysterectomy were eligible for the study. The day before radical hysterectomy we injected technetium^99^m-labelled nanocolloid in each quadrant of the cervix. Dynamic and static images were recorded using a gamma camera. SLNs were identified intraoperatively using a handheld gamma-detection probe. After resection of SLNs a standard radical hysterectomy with pelvic lymph node dissection was performed. Patients and tumour characteristics were compared with sentinel node detection and with final histopathological and immunohistochemical results. Scintigraphy showed focal uptake in 13 of the 14 patients. Intraoperatively we detected 26 sentinel nodes by gamma probe. In 8 of 13 patients, one or more sentinel nodes were identified unilaterally, in 5 women bilaterally. Histologically positive SLNs were found in only 1 patient. We did not find any false-negative SLN in our series. In conclusion identification of sentinel nodes in cervical cancer is feasible with preoperatively administered technetium^99^m-labelled nanocolloid. A larger series will be required to establish sentinel node detection in cervical cancer for further therapy concepts and planning. © 2001 Cancer Research Campaignhttp://www.bjcancer.com


*British Journal of Cancer* (2001) **85**(6), 791–794
© 2001 Cancer Research Campaign
doi: 10.1054/ bjoc.2001.2005, available online at http://www.idealibrary.com on IDEAL

### Sentinel node procedure in Ib cervical cancer: a preliminary series

**T Lantzsch[1], M Wolters[1], J Grimm[2], T Mende[2], J Buchmann[3], G Sliutz[4] and H Koelbl[1]**

Departments of [1]Gynecology, [2]Nuclear Medicine, [3]Pathology, Martin-Luther-University, D-06097 Halle/Saale, Germany; [4]Department of Gynecology, University of Vienna, A-1090 Vienna, Austria

**Summary** The aim of this study was to determine the diagnostic accuracy and feasibility of sentinel lymph node (SLN) detection using a gamma probe in patients with Figo IB cervical cancer. Between January 1999 and September 2000, 14 patients with cervical cancer, planned for radical hysterectomy were eligible for the study. The day before radical hysterectomy we injected technetium$^{99}$m-labelled nanocolloid in each quadrant of the cervix. Dynamic and static images were recorded using a gamma camera. SLNs were identified intraoperatively using a handheld gamma-detection probe. After resection of SLNs a standard radical hysterectomy with pelvic lymph node dissection was performed. Patients and tumour characteristics were compared with sentinel node detection and with final histopathological and immunohistochemical results. Scintigraphy showed focal uptake in 13 of the 14 patients. Intraoperatively we detected 26 sentinel nodes by gamma probe. In 8 of 13 patients, one or more sentinel nodes were identified unilaterally, in 5 women bilaterally. Histologically positive SLNs were found in only 1 patient. We did not find any false-negative SLN in our series. In conclusion identification of sentinel nodes in cervical cancer is feasible with preoperatively administered technetium$^{99}$m-labelled nanocolloid. A larger series will be required to establish sentinel node detection in cervical cancer for further therapy concepts and planning. © 2001 Cancer Research Campaign http://www.bjcancer.com

**Keywords**: sentinel lymph node; cervical cancer; lymphoscintigraphy, lymphatic mapping

Sentinel lymph node status provides important information in patients with tumours that metastasise to superficial lymph nodes, such as melanoma, breast cancer or vulvar cancer (Morton et al, 1992; Giuliano et al, 1994; Levenback et al, 1994). The sentinel lymph node (SLN) is defined as the first draining lymph node of an anatomical region, and the histological examination of the sentinel lymph node would be representative for the non-sentinel lymph nodes. A histologically negative sentinel lymph node would predict the absence of tumour metastases in the other non-sentinel lymph nodes (Morton et al, 1992). The methods to identify such nodes are staining with isosulfan blue dye (Lymphhazurin 1%, Ben Venue Labs, Inc, Richmond, VA) or by detection of the lymph node uptake of a radionuclide that spreads the same way as lymphatics. Detection of SLN has nearly become a standard technique for determining the nodal status in patients with cutaneous melanoma (Statius Muller et al, 1999). The successful identification of the sentinel lymph node by a gamma camera is only the first step, because it is necessary to examine the sentinel nodes histopathologically and immunohistochemically for accurate identification of even microscopic metastasis.

In superficial tumours, such as melanomas or breast cancer, SLN detection can prevent unnecessary extensive lymph node resections. Radical hysterectomy with pelvic lymphadenectomy is the most commonly performed definitive surgical procedure for patients with Figo IB cervical cancer. Few of these patients do have pelvic lymph node metastasis (Delgado et al, 1989; Magrinal et al, 1999), but many patients with radical lymph node resection suffer from side effects after surgery. Therefore some investigators recommended a less radical approach for early cervical cancer with precise preoperative determination of lymph node metastases (Kinney et al, 1995; Shingleton, 1998; Magrina et al, 1999). In spite of computed tomography and magnetic resonance imaging in clinical staging of cervical cancer patients accuracy of preoperative lymph node status is rather poor (Scheidler et al, 1997).

Therefore the aim of the present study was to examine the feasibility and validity of sentinel lymph node detection in patients with early cervical cancer to possibly reduce morbidity following possibly unnecessary radical lymph node resection.

### PATIENTS AND METHODS

Between January 1999 and September 2000 all patients with histologically proven early cervical cancer who were referred to our department for radical hysterectomy, were asked to participate in this study, approved by the local ethics committee. Patients with prior chemotherapy, pelvic radiotherapy or prior retroperitoneal surgery were excluded. Finally 14 patients with cervical cancer stage IBI were eligible for this study and signed written informed consent. Patients age ranged from 28 to 67 years (mean 46 years). All patients underwent preoperative CT and MRI scan. Clinical patient and tumour characteristics are shown in Table 1.

On the day before surgery we intradermally injected one depot of 99m-Tc-labelled human colloid (Albu-res®; Pharmaceutical Nycomed Amersham, Braunschweig, Germany) in each quadrant of the cervix. The colloid was filtered to a range from 200–450 nm to obtain the small particle fraction. The dose ranged from 60–111 MBq. For application we used a 27 gauge hypodermic needle. After injection, dynamic lymphoscintigraphy (28 images, 60

*Received 8 February 2001*
*Revised 3 June 2001*
*Accepted 5 July 2001*

*Correspondence to:* T Lantzsch

**Table 1** Patients characteristics

| | |
|---|---|
| N (Stage IBI) | 14 |
| Age | 28–67 |
| Tumour size (cm) | 1.6–3.8 |
| Histology | |
| SCC | 12 |
| Adenocarcinoma | 2 |
| Grading | |
| I | 5 |
| II | 9 |
| III | 0 |

**Table 2** Sentinel Lymph Node Pathology

| | |
|---|---|
| Sentinel node detection | 13/14 patients |
| Number of SLN | 26 LN |
| Localization of SLN | |
| External iliac | 17 LN |
| Internal iliac | 5 LN |
| Obturator nerve | 3 LN |
| Parametrium | 1 LN |
| Pathology of SLN | |
| Standard staining (H&E) | 2 LN (1 patient) |
| Immunohistochemistry | 2 LN (1 patient) |
| Other LN | 0 LN |

seconds per frame, low-energy high-resolution collimator, matrix $64 \times 64$ or as high as available) was done using a single- or double-headed gamma camera in patients supine position from an anterior view. Additional static images (1 image per 5 minutes) from anterior and lateral views were taken after the dynamic study and about 2 hours after injection. The localization of the sentinel node was marked on the skin to guide the surgeon.

At the time of surgery first the hot nodes were detected before and after opening the retroperitoneal spaces of the pelvic side wall using a hand-held collimated gamma probe (ScintiProbe MR-100, pol.hi.tech, Carsoli, C11 probe, Italy). The count rate in vivo and in vitro was determined. The localization and the count rate of all suspected sentinel nodes in relation to the major pelvic vessels were documented exactly. After separate removal of the radioactive nodes, the complete nodal tissue along the obturator nerve and the iliacal vessels was removed bilaterally up to the level of the aortic bifurcation. Finally, the pelvis was re-examined with the gamma probe to detect residual radioactivity. After sentinel node procedure radical hysterectomy was completed.

Routine histopathological examination of the sentinel and of all other lymph nodes was performed using haematoxylin-eosin staining and serial sections with standard techniques. In case of negative sentinel nodes additional immunohistochemical examination was performed. Findings at surgery were correlated with final pathology reports.

### RESULTS

All 14 patients underwent transperitoneal lymph-node resection at the planned radical hysterectomy. Lymphoscintigraphy showed focal uptake of technetium$^{99}$ in 13 of 14 patients (Figure 1). Only in 1 woman neither lymphoscintigraphy on the day before surgery nor intraoperative gamma probe detection demonstrated focal uptake of technetium$^{99}$. This was due to incorrect subdermal injection and spillage of the tracer, therefore no SLN was identified. Our results are shown in Table 2. We could detect 26 sentinel nodes by handheld gamma probe during surgery. In 8 of 13 patients, one or more unilateral sentinel nodes were identified intraoperatively by the gamma probe, in 5 women we found SLNs bilaterally. The location of SLNs was variable and differed from the external iliac, internal iliac, parametrial and obturator lymph node chains. 25 histologically negative SLNs were retrieved from lymph node chains in which nodal disease was absent in final histology and immunohistochemistry. We found histopathologically positive sentinel lymph nodes in only 1 patient and immunohistochemical examination demonstrated positive results, too. In this case we even found 2 sentinel nodes. The first sentinel node was located in the left parametrium, the second was located at the right external iliac area and both nodes were the only histopathologically positive nodes, accurately representing the real nodal status.

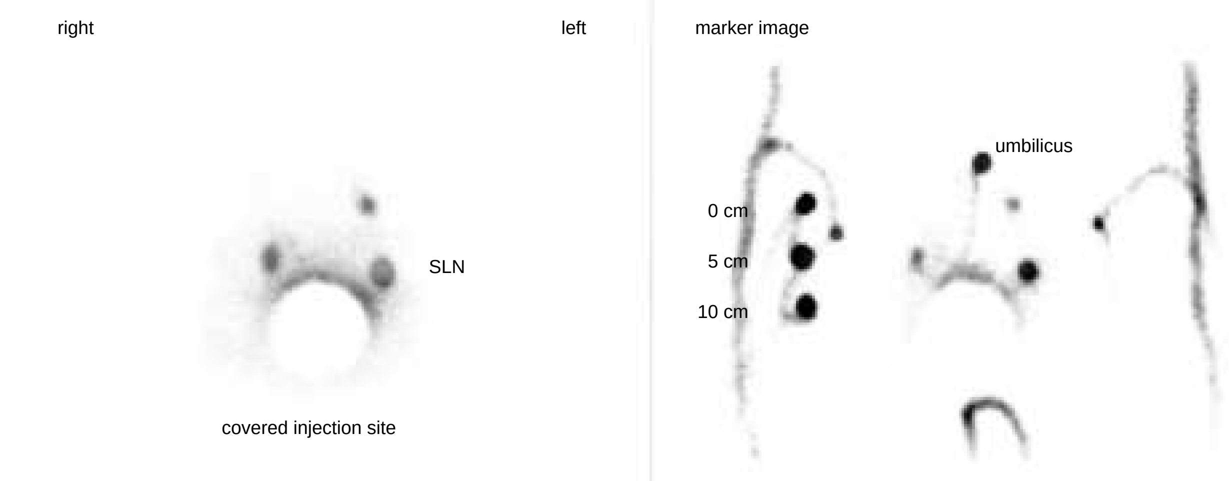

**Figure 1** Lymphoscintigraphy in cervical cancer

*© 2001 Cancer Research Campaign*

**Table 3** Comparison of our results according to literature

| Author | Method | N/patients | SLN found | SLN pathology | SLN neg/other LN pathology | SLN detection rate |
|---|---|---|---|---|---|---|
| Medl et al | Blue dye | 3 | 2–3 per patient | All pos | Neg | 100% |
| Echt et al | Blue dye | 13 | in 2 patients | Not described | Not described | 15.4% |
| O'Boyle et al | Blue dye | 20 | 23 nodes in 12 patients | 3 | 1 | 60% |
| Dargent et al | Blue dye | 35 | 63 nodes in 35 patients | 11 | Neg | 100% |
| Verheijen et al | Scintigraphy | 10 | in 8 patients | 1 | 1 | 80% |
| | (Blue dye) | 10 | in 4 patients | (1) | (1) | 40% |
| Kamprath et al | Scintigraphy | 18 | in 16 patients | 1 | Neg | 88.8% |
| Lantzsch et al | Scintigraphy | 14 | 26 nodes in 13 patients | 2 pos in 1 patient | Neg | 93% |

### DISCUSSION

The validation of the sentinel node procedure as it was introduced in 1977 by Cabanas has led to the rediscovery of lymphoscintigraphy (Cabanas, 1977). The combination of preoperative lymphatic mapping with intraoperative probe detection is increasingly used to identify the sentinel node in melanoma, breast cancer, and in other malignancies such as penile and vulvar cancer. Different studies showed that the sentinel node detection is a highly accurate diagnostic procedure (Giuliano et al, 1994; DeHullu et al, 1998; Gershenwald et al, 1999; DeHullu et al, 2000). In contrary to superficial carcinoma the physiology of lymphatic drainage from the cervix is not yet well established. A technique based on the SLN procedure that makes mapping of the regional lymphatics possible and feasible may be appropriate in this patient collective. In the past there was no evidence for sentinel node stations in cervical cancer. Benedetti-Panici et al examined lymphatic spread through paracervical tissue but found metastases in that area in only 29% (Benedetti-Panici et al, 1996). In cases of lymph node metastases the superficial obturator area was affected most commonly (86%). As a matter of fact radical lymph node dissection during surgery in early stage cervical cancer is discussed controversially (Kinney et al, 1995; Shingleton, 1998; Magrina et al, 1999). For instance, cervical tumour size less than 2 cm has been associated with nodal metastases in only 0–16% of cases (Delgado et al, 1989; Magrina et al, 1999). Recently some authors described the sentinel node procedure in cervical cancer by blue dye uptake (Echt et al, 1999; Dargent et al, 2000; Medl et al, 2000; O'Boyle et al, 2000) or by lymphoscintigraphy (Kamprath et al, 2000), whereas some authors used both techniques to detect SLNs in cervical cancer. Verheijen et al found in a series of 10 patients that the typical location of sentinel nodes was the external iliac area (6 of 18 sentinel nodes) and the chain of the obturator nodes (Verheijen et al, 2000). Verheijen et al also showed that visual detection of dye uptake in lymph nodes is difficult and sometimes unreliable especially in the parametrial nodes. In his study, blue staining was seen in only 4 of 10 women and did not identify any sentinel nodes additionally to those detected by the gamma probe. Echt et al reported a SLN-detection rate for cervical cancer of 15.4% using blue dye (Echt et al, 1999). O'Boyle et al reported a detection rate of 60% in his series of 20 patients using isosulfan blue dye (O'Boyle et al, 2000). Medl et al reported that he found in all 3 cases 2 to 3 blue-stained (sentinel) lymph nodes located either at the external iliac or in the obturator chain. The blue coloured nodes were histopathological positive, whereas all other pelvic lymph nodes removed were negative (Medl et al, 2000). Kamprath et al investigated the validity of laparoscopic SLN detection using lymphoscintigraphy. They detected sentinel nodes in 16 of 18 patients (Kamprath et al, 2000). Dargent et al reported a failure rate between 10% and 50%, depending on the amount of blue dye injected peritumourally (Dargent et al, 2000).

In our present study we evaluated the combination of preoperative lymphatic mapping with intraoperative probe detection in IBI cervical cancer patients. According to the published literature we could show that most SLNs were found in the external iliac and obturator area.

In case of negative sentinel node, we did not find any other histopathologically positive lymph node. According to our results, the necessity of sentinel lymphonodectomy in patients with negative SLNs has to be questioned. However randomised trials are needed for assessing accuracy of this technique. In patients with positive SLNs full lymphonodectomy still is mandatory.

Detection rate compared with previous data in the literature is shown in Table 3. In comparison with the visual detection rates in those studies using blue dye our detection rate and the detection rate in those studies using lymphoscintigraphy was good. If the sentinel node procedure in cervical cancer proves to detect all microscopically involved nodes, treatment of women suffering from cervical cancer could be modified to less, or if necessary to even more radical, treatment. In patients with Figo IB cervical cancer complete lymphonodectomy might be omitted if the sentinel lymph node is negative. Although sentinel node detection by lymphoscintigraphy requires special equipment and is rather expensive it could help us to save money and time in the operating theatre.

### REFERENCES

Benedetti-Panici P, Maeschi F, Scambia G, Greggi S, Cutillo G and d'Andrea G (1996) Lymphatic spread of cervical cancer: An anatomical and pathological study based on 225 radical hysterectomies with systematic pelvic and aortic lymphadenectomy. *Gynecol Obstet* **62**: 19–24, doi: 10.1006/gyno. 1996.0184

Cabanas RM (1977) An approach for the treatment of penile carcinoma. *Cancer* **39**: 456–466

Dargent D, Martin X and Mathevet P (2000) Laparoscopic assessment of the sentinel lymph node in early stage cervical cancer. *Gynecol Oncol* **79**: 411–415, doi: 10.1006/gyno.2000.5999

De Hullu JA, Doting E, Piers DA, Hollema H, Aalders JG, Koops HS, Boonstra H and van der Zee AG (1998) Sentinel lymph node identification with Technetium99m-labeled nanocolloid in squamous cell cancer of the vulva. *J Nucl Med* **39**: 1381–1385

De Hullu JA, Hollema H, Piers DA, Verheijen RHM, van Diest PJ, Mourits MJE, Aalders JG and van der Zee AGJ (2000) Sentinel lymph node procedure is highly accurate in squamous cell carcinoma of the vulva. *J Clin Oncol* **18**: 2811–2816

Delgado G, Bundy BN, Fowler WC Jr, Stehman FB, Sevin B, Creasman WT, Major F, DiSaia P and Zaino R (1989) A prospective surgical pathological study of

*© 2001 Cancer Research Campaign* 

stage I squamous carcinoma of the cervix: A Gynecologic Oncology Group Study. *Gynecol Oncol* **35**: 314–320

Echt ML, Finan MA, Hoffmann MS, Kline RC, Roberts WS and Fiorica JV (1999) Detection of sentinel lymph nodes with lymphazurin in cervical, uterine, and vulvar malignancies. *South Med J* **92**: 204–208

Gershenwald JE, Thompson W, Mansfield, Lee JE, Colome MI, Tseng CH, Balch CM, Reintgen DS and Ross MI (1999) Multi-institutional melanoma lymphatic mapping experience: The prognostic value of sentinel lymph node status in 612 stage I or II melanoma patients. *J Clin Oncol* **17**: 976–983

Giuliano AE, Kirgan DM, Geunther JM and Morton DL (1994) Lymphatic mapping and sentinel lymphadenectomy for breast cancer. *Ann Surg* **220**: 391–401

Kamprath S, Possover M and Schneider A (2000) Laparoscopic sentinel node detection in patients with cervical cancer. *Am J Obstet Gynecol* **6**: 1648

Kinney WK, Hodge DO, Egorshin EV, Ballard DJ and Podratz KC (1995). Identification of a low-risk subset of patients with stage IB invasive squamous cancer of the cervix possibly suited to less radical surgical treatment (see comments). *Gynecol Oncol* **57**: 3–6, doi:10.1006/gyno.1995.1091

Levenback C, Burke TW, Gershenson DW, Morris M, Malpica A and Ross MI (1994) Intraoperative lymphatic mapping for vulvar cancer. *Obstet Gynecol* **84**: 163–167

Magrina JF, Goodrich MA, Lidner TK, Weaver AL, Cornella JL and Podratz KC (1999) Modified radical hysterectomy in the treatment of early squamous cervical cancer. *Gynecol Oncol* **72**: 183–186, doi:10.1006/gyno.1998.5245

Medl M, Peters-Engl C, Schütz P, Vesely M and Sevelda P (2000) First report of lymphatic mapping with isosulfan blue dye and sentinel node biopsy in cervical cancer. *Anticancer Research* **20**: 1133–1134

Morton DL, Wen DR, Wong JH, Economou JS, Cagle LA and Storm FK (1992) Technical details of intraoperative lymphatic mapping for early stage melanoma. *Arch Surg* **127**: 392–399

O'Boyle JD, Coleman RL, Bernstein SG, Lifshitz S, Muller CY and Miller DS (2000) Intraoperative lymphatic mapping in cervix cancer patients undergoing radical hysterectomy: a pilot study. *Gynecol Oncol* **79**: 238–243, doi:10.1006/gyno.2000.5930

Scheidler J, Hricak H, Yu KK, Subak L and Segal MR (1997) Radiological evaluation of lymph node metastases in patients with cervical cancer. A meta-analysis. *JAMA* **278(13)**: 1096–1101

Shingleton HM (1998) Surgery for cervical cancer: A time for reassessment (see comments). *Gynecol Oncol* **69**: 8–13, doi:10.1006/gyno.1998.4957

Statius Muller MG, van Leeuwen PAM and Borgstein PJ (1999) The sentinel node procedure in cutaneous melanoma. An overview of 6 years experience. *Eur J Nucl Med* **26**: 20–25

Verheijen FHM, Pijpers R, van Diest PJ, Burger CW, Buist MR and Kenemans P (2000) Sentinel node detection in cervical cancer. *Obstet Gynecol* **96**: 1315–138

 *© 2001 Cancer Research Campaign*
